# Genetics and Epigenetics of Chemoinduced Oral Mucositis in Paediatric Patients with Haematological Malignancies—A Review

**DOI:** 10.3390/epigenomes9020016

**Published:** 2025-05-30

**Authors:** Juliana Ramalho Guimarães, José Maria Chagas Viana Filho, Naila Francis Paulo de Oliveira

**Affiliations:** 1 Graduate Program in Dentistry, Health Sciences Center, Federal University of Paraíba—UFPB, João Pessoa 58051-900, PB, Brazil; juliana.guimaraes@academico.ufpb.br; 2 Faculty of Dentistry of Acorverde, University of Pernambuco—UPE, Arcoverde 56503-146, PE, Brazil; viana.filho5@gmail.com; 3 Department of Molecular Biology, Center for Exact and Natural Sciences, Federal University of Paraíba—UFPB, João Pessoa 58051-900, PB, Brazil

**Keywords:** oral mucositis, polymorphism, DNA methylation, children, genetic, epigenetic, leukaemia, lymphoma

## Abstract

Background: Oral mucositis (OM) is a painful inflammation resulting from chemotherapy. It is dependent on factors such as age, gender, chemotherapy regimen, oral health, immunological and nutritional status, and genetics. Objectives: The aim of the study was to conduct a narrative review to compile studies on the contribution of genetic and epigenetic aspects to the pathogenesis of OM in children with haematological malignancies undergoing chemotherapy treatment. Methods: The literature search was performed in Pubmed, Scopus, Web of Science, Cochrane, Lilacs, and grey literature databases covering articles published since 2010. Results: Twenty-two studies investigating polymorphisms and four studies investigating DNA methylation were included. Polymorphisms in the *MTHFR*, *ABCB1*, *ABCC2*, *ABCG2*, *SLCO1B*, *miR-1206*, *miR-3683*, *CAT*, and *VDR* genes were associated as risk factors for OM and polymorphisms in the *TYMS* and *miR-4268* genes were associated as protective factors. With regard to DNA methylation, associations such as protection or susceptibility to OM have not yet been proven. However, studies have shown that *DNMT1* methylation and hypomethylation in total DNA and in the *TNF-α* gene are associated with recovery of the oral mucosa. Conclusions: Genetic variants are associated with OM in various biological pathways, such as folate metabolism, transport proteins, epigenetic machinery, oxidative stress, and vitamin D metabolism. The DNA methylation profile, which is still poorly understood in the pathogenesis of OM, is associated with mucosal recovery (inflammation and epigenetic machinery). Genetic and epigenetic markers may be tools to indicate a patient’s susceptibility to developing OM, and epigenetic markers may be a target for therapies.

## 1. Introduction

The introduction of chemotherapy in 1940 has undoubtedly increased the chances of survival. Although targeted drugs and biological agents have achieved excellent results, cytotoxic chemotherapy remains an important component of cancer treatment. Chemotherapy has side effects due to its effect on normal tissues with high mitotic power, such as the oral mucosa [1,2].

Oral mucositis (OM) is an inflammatory reaction of the tissues of the oral cavity to the direct therapeutic toxicity of drugs. These lesions are more frequent and severe in children due to the intense mitotic activity at this stage and are frequently observed in patients with haematological tumours treated with methotrexate (MTX) [1,3].

Clinically, the changes can range from redness and oedema to confluent ulceration throughout the mucosa. The most severe manifestations of oral mucositis can cause serious harm to the patient, such as severe pain, difficulty in speaking, impaired nutritional status, bleeding, myelosuppression, and sepsis, limiting the patient’s quality of life and, more drastically, death has been observed as a result of the developmental clinic and complications of severe oral mucositis. In addition, oral mucositis can lead to treatment discontinuation and compromise the effectiveness of cancer treatment. Thus, oral mucositis prolongs patient suffering and hospital stay and increases costs [1,2,4].

A pathobiological model with multiple mechanisms has been proposed to explain the occurrence of oral mucositis and its gradation at the molecular level. It can be arbitrarily divided into five phases according to the biological processes, which are (A) initiation, (B) message generation, (C) signal amplification, (D) ulceration, and (E) healing [1,5].

The diagnosis of oral mucositis and the grading of its severity can be made using some tools, such as the modified Oral Assessment Guide (OAG)—grade 1 to 3, the World Health Organisation scale (WHO)—grade 0 to 4 and the National Cancer Institute’s—Common Terminology Criteria for Adverse Events (NCI-CTCAE)—grade 0 to 5. These scales assess objective (erythema, ulceration) and subjective (pain, difficulty speaking, and eating) signs of oral mucositis (Table 1) [6,7,8].

There are large interindividual differences in susceptibility to the development of oral mucositis. The development of mucositis depends not only on the cancer treatment regimen, dosage and number of cycles, but also on patient characteristics such as age, gender, and genetic factors [1,2,3,5].

Mendel’s principles (1865), the isolation of the DNA molecule (1869), and the resolution of the DNA double helix (1959) established the principles of genetics and heredity [9]. Genetics deals with genes that consist of DNA sequences that code for RNA. These DNA sequences can vary from individual to individual and when this variation occurs with a frequency of more than 1% in the population, they are called genetic polymorphisms (or genetic variants) [10,11,12]. Genetic polymorphisms involve changes in the sequence of bases in DNA, either by insertion, deletion, copy number variants, inversions, or substitution of nucleotides [9,10]. The biological effect of genetic polymorphisms (changes in gene expression or protein activity) is associated with disease susceptibility or protection and can, therefore, be used to predict disease risk [11,12].

Conrad Waddington (1957) coined the term epigenetics to summarise the connection between gene and environment [13]. Epigenetics studies the chemical modifications of DNA and histone proteins as well as non-coding RNAs. Epigenetic mechanisms do not change the sequence of DNA bases and are involved in the control of gene expression [14,15]. DNA methylation is the best-studied epigenetic mechanism and consists of a covalent modification of DNA in which a methyl group (CH_3_) generated in the folate cycle is transferred to cytosines upstream of guanines (CpG dinucleotides) by DNA methyltransferases (DNMTs). These dinucleotides are mainly located in the regions of the gene promoters, and the presence of the CH_3_ radical inhibits the binding of transcription factors in these regions. The lack of binding of transcription factors to their specific sites can result in the absence of or decrease in gene transcription [15]. A striking feature of epigenetic mechanisms is that they are reversible and can vary depending on gender, age, and environmental factors such as individual habits, infections, radiation, medication, etc. [15]. In addition to genetic polymorphisms, changes in the DNA methylation profile are also associated with a variety of diseases [14].

The literature shows that genetic and epigenetic marks are associated as risk or protective factors for diseases. Genetic marks can lead to changes in gene expression or protein activity, while epigenetic marks are associated with changes in gene expression [11,12,14,15]. As epigenetic marks are reversible, they can also be targets for treatment [15]. Both are expected to contribute to the development of multifactorial diseases, and the same is expected for oral mucositis. These data are tools for personalised diagnoses and treatments, which are, therefore, more accurate and effective [16].

This narrative review, therefore, examines the contribution of genetic and epigenetic markers to the pathogenesis of oral mucositis in paediatric patients with haematological malignancies in order to collate these studies and propose new ones.

## 2. Methodology

Studies investigating the association between genetic or epigenetic markers and oral mucositis in paediatric patients with haematological malignancies undergoing chemotherapy treatment were the objective of this review. The literature search was conducted in Pubmed, Scopus, Web of Science, Cochrane, Lilacs, and grey literature databases and included articles published since 2010. The inclusion criteria were as follows: observational studies, case–control studies, or cohort analytical studies published in Portuguese, Spanish, or English. The exclusion criteria comprised literature reviews, book chapters, letters to the editor, and case reports.

## 3. Genetics and Oral Mucositis

The genetic polymorphisms that have been studied in the context of oral mucositis in children with haematological malignancies can be divided into four biological pathways: folic acid metabolism, transport proteins, epigenetic machinery and other pathways such as inflammation, vitamin D metabolism, oxidative stress, and others. They are listed in Table 2 in the order of publication [17,18,19,20,21,22,23,24,25,26,27,28,29,30,31,32,33,34,35,36,37,38].

### 3.1. Polymorphisms in the Folic Acid Metabolism Pathway

The folic acid cycle (also known as folate cycle) is crucial for DNA synthesis and repair, the biosynthesis of amino acids, the conversion of homocysteine to methionine, and DNA and RNA methylation reactions. MTX impairs the folate cycle as it is a folate antagonist. Therefore, abnormal functions of the enzymes involved in this cycle due to genetic alterations contribute to adverse reactions to chemotherapy, such as oral mucositis [39].

The *MTHFR* gene encodes the enzyme methylenetetrahydrofolate reductase, which plays a central role in the folate cycle. Almost half of the articles included in this review dealt with this gene (10 articles). The CT/TT genotypes of the rs1801133 polymorphism were associated with oral mucositis in Egyptian, Chinese, and Slovenian children [17,19,32,34], while the CC genotype was associated with mucositis in Mexican children [25]. In contrast, no association was found in Serbian and Brazilian children and in other Chinese studies [18,30,31,33,35]. The differences between the studies can be explained by the following factors: treatment phase (some studies mention the consolidation phase, others do not), study design (one study delineated the groups according to MTX excretion capacity), sample size, ethnicity, and treatment protocols (although all used MTX, the different protocols contain different doses and different agents than MTX).

The polymorphism rs1801133 (also known as C677T) is a single nucleotide polymorphism (SNP) in which the C nucleotide is replaced by the T nucleotide at position 677 of exon 4; this leads to an amino acid exchange from alanine to valine in the protein (Ala222Val), reducing enzyme activity. As a result, mutant individuals (CT and TT) have 60 and 30% of normal MTHFR activity, respectively. Decreased MTHFR activity may lead to higher intolerance to MTX [40].

The second most studied polymorphism was rs1801131 (also known as A1298C), which also leads to decreased *MTHFR* activity was not associated with OM [17,19,30,31,32,34]. The SNP rs2274976 was also not associated with OM [32].

Other folic acid pathway genes, such as MTRR, TYMS, MTHFD1, MTR, MS, FPGS, GGH, and DHFR were not associated as a risk factor for OM in Slovenian, Chinese, Dutch, and Serbian children [19,21,28,30,31,32]. A study showed that the 3R3R genotype of the *TYMS* gene (rs34743033) was associated as a protective factor for OM in Slovenian children [19]. The rs34743033 polymorphism is a double or triple tandem repeat polymorphism of 28 base pairs in the 5′-UTR region (untranslated region), and the 3R3R genotype is associated with increased *TYMS* expression [41]. Interestingly, one study showed, although without statistical significance, that Dutch children with the 2R2R genotype in combination with the 2R/3RC and 3RC/3RC genotypes of another tandem polymorphisms in the same gene (rs2853542) associated with lower *TYMS* expression were more likely to develop mucositis [28].

### 3.2. Polymorphisms in the Transport Proteins Pathway

Genes that encode transport proteins, such as solute carrier (*SLC*) family and the ATP-binding cassette (*ABC*) family, are also involved in OM in children. MTX possibly enters via the solute carriers and exits via the ABC transporters [41].

The *SLC* family genes studied comprise: *SLC19A1*, *SLC28A2*, *SLCO1A2*, and *SLCO1B*. The rs1051266 polymorphism in the *SLC19A1* gene was addressed in four articles and no association with OM was found [19,26,30,31]. The rs2306283 polymorphism in the *SLCO1B1* gene was discussed in 2 Chinese articles [26,31] and was associated with OM in one of the studies in which the children had the genotype AG/GG [31]. The *SLCO1B1* gene encodes the solute carrier organic anion transporter family member 1B1, which transports endogenous and exogenous organic solutes. The rs2306283 polymorphism (also known as A388G) is a SNP in which the A nucleotide is replaced by the G nucleotide at position 388 of exon 5, resulting in an exchange of asparagine for aspartate amino acids in the protein (Asn130Asp), which may reduce transporter activity. It is speculated that the decrease in the activity of this transporter, i.e., the decrease in the entry of MTX by SLCOB1, is associated with a delay in the elimination of MTX and consequently an increase in its concentration in plasma and higher rates of toxic events such as mucositis [31,41]. Other polymorphisms were investigated in these genes and no association with OM was found [19,26,30,31,32,33].

The *ABC* family genes studied comprise: *ABCB1*, *ABCC1*, *ABCC2*, *ABCC4*, *ABCC5*, and *ABCG2*. The rs1045642 polymorphism in the *ABCB1* gene was covered in five studies [20,25,31,32] and was associated with OM in Turkish children who had the CT genotype [20] and in Chinese children who had the CT/TT genotypes [32]. The *ABCB1* gene (also known as *MDR1*) encodes the ATP-binding cassette of subfamily B, member 1, which is a drug efflux pump. The rs1045642 polymorphism is a SNP (also known as C3435T) in which the C nucleotide is replaced by the T nucleotide at position 3435 of exon 26. It is a synonymous SNP, as the base substitution still codes for isoleucine (Ile1145Ile), but appears to be associated with reduced transporter activity [42]. It is possible that children carrying the T allele have a lower rate of MTX efflux and, therefore, this agent remains in the cell longer and can cause toxic events such as oral mucositis. Another SNP in the *ABCB1* gene, rs1128503, was also associated with OM in Chinese children carrying the CT/TT genotypes [32], but the same was not observed in other Chinese studies [26,31]. The SNP rs1128503 (also known as C1236T) corresponds to the substitution of the nucleotide C by T at position 1236 of exon 12, resulting in a synonymous SNP (GlyGly). However, experiments have shown that this alters the conformation of the protein and leads to reduced substrate specificity [43], which could result in delayed MTX excretion. The rs717620 SNP in the *ABCC2* gene has been discussed in four articles [22,31,32,35] and was associated with OM in a Chinese study in which the children had the CT/TT genotypes [22]. The *ABCC2* gene encodes ATP-binding cassette subfamily C member 2 and the rs717620 polymorphism (also known as C-24T) is a SNP in which the C nucleotide is replaced by the T nucleotide at position 21 of the promoter, resulting in a decrease in *ABCC2* expression [22,41]. It is possible that children with the T allele have lower expression of *ABCC2*, and this is related to the lower capacity for MTX efflux, resulting in MTX remaining in the cell longer and causing toxic effects. The SNP rs2231142 in the *ABCG2* gene was discussed in two articles [32,35] and was associated with OM in Brazilian children who had the CA genotype [35]. The *ABCG2* gene encodes the ATP-binding cassette of subfamily G, member 2, and SNP rs2231142 (also known as C421A) corresponds to the substitution of nucleotide C by A in position 421 of exon 5, resulting in an exchange of glutamine amino acids for lysine in the protein (Gln141Lys), causing a structural and functional defect of the transporter [35,41]. It is possible that children carrying the A allele have a lower MTX efflux rate and, therefore, this drug stays in the cell longer and can cause toxic events such as oral mucositis. Other polymorphisms in this gene family have also been studied, but no association with OM was found [19,25,26,31].

### 3.3. Polymorphisms in the Epigenetic Machinery Pathway

Genes involved in the epigenetic machinery, such as *miR* (micro-RNAs), genes involved in the biogenesis and processing of miRNA and *DNMTs* (DNA methyltransferases), have also been studied. Micro-RNAs inhibit translation of target genes and DNA methyltransferases add the methyl radical to DNA [10,15]. Genetic alterations in epigenetic genes can lead to changes in gene expression, which in turn can contribute to oral mucositis.

The rs2114358 polymorphism in the *miR*-1206 gene was associated with OM in Dutch children who had the GG genotype [27]. The miR-1206 gene encodes microRNA-1206 and the polymorphism rs2114358 corresponds to the exchange of the nucleotide G for A. This change leads to a change in the secondary structure. In silico analyses have shown that the GG genotype is associated with greater stability of the molecule, which may lead to increased expression of *miR-1206* and a reduction in its targets. The same analysis revealed that some of its targets are genes encoding membrane transporters and enzymes involved in folic acid metabolism, as mentioned earlier, e.g., *SLCO1A2*, *ABCC2*, *ABCG2*, *TYMS*, *FPGS* genes [27]. The rs6977967 polymorphism in the miR-3683 gene was associated with OM in Spanish children who had the GG genotype [29]. The *miR-3683* gene encodes microRNA-3686 and the rs6977967 polymorphism corresponds to the exchange of the nucleotide T for C. In silico analyses revealed no changes in secondary structure when the bases were altered. In addition, the *SHMT1* gene, which is involved in MTX metabolism, was found to be a target of micro-RNA-3686 [29]. In Spanish children, the rs4674470 polymorphism in the *miR-4268* gene was a protective factor for OM in children with genotypes AG/GG [29]. No association was found with OM in genes involved in the biogenesis and processing of miRNA [23] and genes encoding DNA methyltransferases [35,38].

### 3.4. Polymorphisms in the Others Pathways

The other genes studied were genes involved in purine metabolism (*XO*, *ATIC*), cell cycle (*CCND1*), transcriptional regulator (*CNOT4*), detoxification (*GSTP1*), inflammation (*IL6* and *TNF-α*), oxidative stress (*SOD* and *CAT*), and vitamin D receptor (*VDR*).

The rs7943316 polymorphism in the gene *CAT* was associated with oral mucositis in Brazilian children who had the genotype AA [36]. The *CAT* gene encodes the antioxidant enzyme catalase, and the rs7943316 polymorphism is a SNP (also known as -21 A/T) in which the A nucleotide is replaced by the T nucleotide at position 21 of the promoter, resulting in decreased enzyme expression. Interestingly, the children with the genotype that might be associated with greater *CAT* expression were the ones who had the severe form of mucositis. The rs1544410 and rs2228570 polymorphisms in the *VDR* gene were associated with oral mucositis in Brazilian children who had the G allele and the CT genotype, respectively [37]. The *VDR* gene encodes the vitamin D receptor, and the rs1544410 polymorphism is a SNP (also known as *Bsm*I) in which nucleotide A is replaced by G at position 63980 in intron 8 and is associated with the regulation of gene expression, particularly mRNA stability. It was suggested that the G allele is associated with reduced *VDR* expression, which in children with mucositis may be related to a reduced capacity to defend against inflammatory processes and may lead to greater tissue damage. The rs2228570 polymorphism is a SNP (also known as *Fok*I) in which the C nucleotide at position 30920 at the junction of intron 1 and exon 2 is replaced by a T, resulting in an additional start codon. The T allele produces a larger protein with lower affinity for the transcription factor TFIIB, representing a transcriptionally less potent VDR protein. Other polymorphisms showed no association with oral mucositis [24,27,31,32,36].

## 4. Epigenetics and Oral Mucositis

Compared to genetic polymorphisms, the epigenetics of oral mucositis has been little studied and only a few biological pathways have been investigated. So far, studies are limited to genes of the epigenetic machinery and pathways of inflammation, oxidative stress and vitamin D metabolism, and they all involve DNA methylation studies. They are listed in Table 3 in order of publication [37,38,44,45,46].

### 4.1. DNA Methylation in the Epigenetic Machinery Pathway

For epigenetic machinery, the targets studied were global methylation and site-specific methylation in genes from the family that encodes DNA methyltransferases (*DNMT1*, *DNMT3A*, *DNMT3B*) and genes from the micro-RNA 9 family (*miR-9-1* and *miR-9-3*).

In the study of global methylation in blood cells of children from the Netherlands, no association with oral mucositis was found in patients analysed before and after chemotherapy with MTX. However, an increase in the level of global methylation (LINE -1 regions) was found after chemotherapy [44].

In the studies of specific sites of *DNMTs* genes, methylation of *DNMT1* was found in the oral mucosa of Brazilian children who had recovered from OM [38]. The *DNMT1* gene encodes DNA methyltransferase 1, which is responsible for maintaining the DNA methylation profile during the cell cycle. Methylation of *DNMT1* may be accompanied by a decrease in its expression, leading to a decrease in global methylation and an increase in gene expression during the healing process, which involves successive cell divisions, cell differentiation and regulation of the inflammatory response [47]. A study in mice showed that the lack of dnmt1 was associated with improved macrophage motility and wound healing [48]. For the *miR-9* family, no associations with OM were found in oral cells of Brazilian children [46].

### 4.2. DNA Methylation in the Inflammation, Oxidative Stress and Vitamin D Metabolism Pathways

The genes covered in these pathways were: interleukin-6 (*IL6*), tumour necrosis factor-alpha (*TNF-α*), catalase (*CAT*), superoxide dismutase (*SOD*), and vitamin D receptor (*VDR*). 

Hypomethylation of *TNF-α* was detected in the oral mucosa of Brazilian children who had recovered from OM [45]. The *TNF-α* gene encodes the pro-inflammatory cytokine tumour necrosis factor-alpha, which plays an important role in the development of inflammation. Hypomethylation in the *TNF-α* promoter may be associated with increased expression of this cytokine and enhancement of tissue damage. The authors discussed the possibility that demethylation occurs during acute inflammation and that this profile can only be observed after mucosal recovery. Interestingly, however, one study has shown that TNF-α is important for the healing of bone fractures and excessive or insufficient levels can hinder the healing process [49]. Thus, this cytokine is observed to play a role in the development of OM, as has already been demonstrated in the literature [1,5], but it may also be important in the healing process. No association with oxidative stress genes or Vitamin D metabolism was found [37,45].

## 5. Biological Pathways Associated with Oral Mucositis

In summary, the biological signalling pathways investigated, their respective targets and the populations addressed are listed in Table 4. 

**Figure 1:**
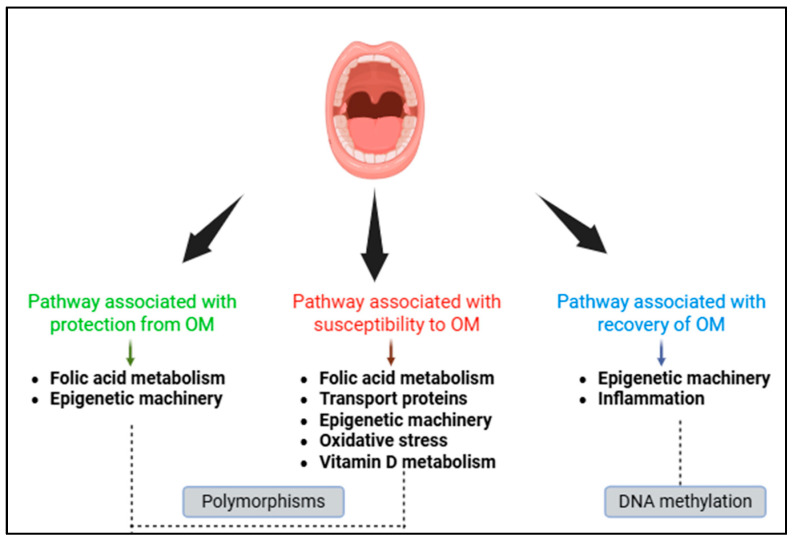
Biological pathways involved in oral mucositis (OM). Pathways leading to protection or susceptibility are supported by genetic data (polymorphisms). Pathways involved in mucosal recovery are supported by epigenetic data (DNA methylation).

The biological pathways associated with the protection, susceptibility and recovery of oral mucositis are summarised in Figure 1.

A total of two polymorphisms were associated as protective factors and 11 as risk factors. The pathway most frequently associated with protective or risk factors was the folic acid pathway, accompanied by transport proteins. Together, these signalling pathways are involved in the entry and exit of MTX from the cell as well as its metabolism within the cell [41,50]. MTX is an antifolate agent that is highly associated with the development of oral mucositis [1,3,30]. Genes involved in the epigenetic machinery are in turn involved in mucosal protection, susceptibility, and recovery. This shows that epigenetic genes are quite important in the context of oral mucositis and these mechanisms deserve to be further explored both in the study of polymorphisms in these genes and in their epigenetic profile.

### 5.1. Pathways Associated with Protection from Oral Mucositis

Protection against OM has been found to be associated with polymorphisms in genes involved in the folate metabolic pathway (*TYMS* gene) and the epigenetic machinery (*miR-4268* gene) [19,29].

The 3R allele of the rs34743033 polymorphism in the *TYMS* gene favours mRNA expression and, thus, enzymatic activity. As this enzyme is a target of MTX, higher TYMS levels favour the metabolism of MTX and reduce its toxicity [50].

The G allele of the rs4674470 polymorphism in the *miR-4268* gene could lead to higher expression of *miRNA-4268* and consequently to lower expression of its target genes, such as *PLD*-related genes [29]. PLD (phospholipase D) modulates pro-inflammatory gene expression and plays an important role in the secretion of cytokines [51], a mechanism involved in the development of mucositis [1,5]. Silencing of *PLD* by microRNAs, therefore, reduces cytokine production [52] and improves mucosal inflammation.

In summary, the rs34743033 and rs4674470 polymorphisms can lead to reduced MTX toxicity and reduced cytokine expression, which explains their protective role in oral mucositis.

### 5.2. Pathways Associated with Susceptibility to Oral Mucositis

Susceptibility to OM has been found to be associated with polymorphisms in genes involved in the folic acid metabolic pathway (*MTHFR* gene), transport proteins (*SLCO1B1*, *ABCB1*, *ABCC2*, and *ABCG2* genes), epigenetic machinery (*miR-1206* and *miR-3683* genes), oxidative stress (*CAT* gene) and vitamin D metabolism (*VDR* gene) [17,19,20,22,25,27,29,31,32,34,35,36,37].

The T allele of the rs1801133 polymorphism of the *MTHFR* gene leads to reduced enzymatic activity. Since this enzyme is a target of MTX, reduced enzymatic activity may mean reduced MTX metabolism, which increases the toxicity of MTX and increases plasma homocysteine levels [40,41,50]. In turn, higher homocysteine levels are associated with inflammation [53].

The G allele of the rs2306283 polymorphism in the *SLCO1B1* gene is associated with reduced activity of this transporter, which promotes the entry of MTX into the cell. This could, therefore, lead to reduced entry of MTX, resulting in delayed elimination and consequently increased plasma concentration and greater toxicity [31]. The T allele of the rs1045642 polymorphism in the *ABCB1* gene is associated with reduced activity of this transporter, which promotes the exit of MTX from the cell. As a result, the drug remains in the cell longer due to the lower excretion rate and causes greater toxicity [41,50]. The T allele of another polymorphism in the *ABCB1* gene, rs1128503, is associated with a lower affinity for its substrate, which in turn may also reduce MTX efflux rates, leading to greater toxicity [41,43,50]. The T allele of the rs717620 polymorphism in the *ABCC2* gene is associated with decreased expression of this transporter, which promotes the exit of MTX from the cell, which in turn may also decrease MTX efflux rates, leading to greater toxicity [41,50]. The A allele of the rs2231142 polymorphism in the *ABCG2* gene is associated with a structural and functional defect in this transporter, which can lead to a reduction in the rate of MTX elimination from the cell and, thus, to greater toxicity [41,50].

The GG genotype of the rs2114358 polymorphism of the *miR-1206* gene is associated with changes in the secondary structure of this microRNA, and in silico analyses show that this structure is associated with greater stability, which may lead to increased expression. Increased expression of *miR-1206* in turn leads to decreased expression of its targets, some of which in silico analyses have shown to be: *SLC* and *ABC* family genes, which, as previously mentioned, act as transporters of MTX into or out of the cell. Reduced expression of these genes affects the entry or exit of MTX into the cell, which can lead to increased toxicity [27]. Another target is the *TYMS* gene, which has already been described above as potentially protective when present at higher concentrations [19,27]. However, as it is a target of *miR-1206*, it may be less expressed and thus contribute to oral mucositis. The GG genotype of the rs6977967 polymorphism of the *miR-3683* gene is not associated with changes in its structure according to in silico analyses, and although it targets genes are involved in MTX metabolism and inflammation, the authors note that they could not explain the effect of this polymorphism on the development of oral mucositis [29].

The AA genotype of the rs7943316 polymorphism in the *CAT* gene is associated with increased expression of the enzyme that neutralises reactive oxygen species (ROS). Interestingly, this genotype has been associated with the development of oral mucositis [36]. The mechanism is difficult to explain, as, in general, a greater ability to neutralise ROS has a protective effect against disease [54]. However, these data cannot be ignored as this genotype, as well as higher enzyme activity, has been associated with other diseases such as asthma and diabetes mellitus type 1 and 2 [55]. For oral mucositis in particular, we can speculate that lower ROS levels hinder the defence against oral pathogens, which in turn contributes to the development of oral mucositis [1,5,54].

The G allele of the rs1544410 polymorphism and the CT genotype of the rs2228570 polymorphism in the *VDR* gene are associated with lower gene expression of the vitamin D receptor and a less potent receptor in recognising its target (transcription factor TFIIB), respectively. The vitamin D–VDR complex can regulate hundreds of genes (up or down), including itself, and it is hypothesised that the presence of alterations at the level or in the structure of these receptors can deregulate the inflammatory process and exacerbate inflammatory responses [56].

In summary, the polymorphisms rs1801133, rs2306283, rs1045642, rs1128503, rs717620, rs2231142, rs2114358, rs6977967, rs7943316, rs1544410, and rs2228570 may lead to increased toxicity of MTX, may decrease the rate of MTX entry and exit from the cell, and lead to decreased expression of genes involved in MTX metabolism, possibly hindering defence against oral pathogens and altering the expression of genes involved in the inflammatory process, which would explain their role in susceptibility to oral mucositis.

### 5.3. Pathways Associated with Recovery of the Oral Mucosa

Mucosal recovery after mucositis was related to the DNA methylation profile in the epigenetic machinery (*DNMT1* gene) and inflammation (*TNF-alpha* gene) as well as in total DNA.

Methylation of the *DNMT1* gene in oral mucosal cells has been associated with mucosal recovery [38]. *DNMT1* encodes the enzyme DNA methyltransferase 1, which acts by incorporating methyl radicals into DNA. DNA methylation is associated with reduced gene expression. In this sense, a possible reduction in DNMT1 levels due to DNA methylation may lead to a reduction in the methylation profile of a variety of genes and consequently to an increase in their expression. Indeed, the literature shows that the wound healing is associated with changes in the methylation profile and gene expression [47,57].

Hypomethylation of the *TNF-α* gene in the oral mucosa was also associated with mucosal recovery [45]. The *TNF-α* gene codes for tumour necrosis-factor alpha, a pro-inflammatory cytokine, and the decrease in the methylation profile of this gene is associated with an increase in its expression [58]. However, the authors found no differences in the oral mucosa during the active phase of inflammation but only, after mucositis. These data show the importance of this gene in mucosal recovery and this hypothesis can be supported by other studies. This cytokine has been shown to be important in the healing of bone fractures where excessive or insufficient levels have impaired the healing process [49]. In addition, blockade of TNF-alpha by monoclonal antibodies has delayed the healing of the oral mucosa after the development of an ulcer [59].

A reduced global DNA methylation profile in oral mucosal cells was associated with mucosal recovery [46]. As mentioned above, changes in the DNA methylation profile are necessary for tissue recovery, as the expression of a variety of genes involved in cell division and wound healing must be re-modulated during this phase [47,57].

In summary, the above data seem to be Consistent, I.E., *DNMT1* methylation, possibly associated with reduced expression, may lead to hypomethylation of *TNF-alpha* and at the global DNA level. This hypothesis is quite plausible as these data come from studies with the same population [38,45,46].

## 6. Final Considerations and Future Perspectives

In short, it was observed that genetic polymorphisms in different biological pathways are associated with OM as a risk or protective factor (Table 4 and Figure 1). Polymorphisms in the *MTHFR*, *ABCB1*, *ABCC2*, *ABCG2*, *SLCO1B*, *miR-1206*, *miR-3683*, *CAT*, and *VDR* genes were associated as risk factors for OM and polymorphisms in the *TYMS* and *miR-4268* genes were associated as protective factors. In some cases, the same polymorphism analysed in different studies did not show the same association with oral mucositis. The differences can be explained by ethnicity, sample size, chemotherapy regimen (agents and dose), treatment phase (induction, consolidation or maintenance), oral health, immunological, and nutritional status of the patient and haematological parameters. Another point that needs to be emphasised is the scale for the diagnosis of OM. Differences in the classification of mucositis may lead to different results between studies [1,2,3,4,5].

One difficulty in these studies is that haematological malignancies are a rare disease and, moreover, OM does not affect all cancer patients, so it is often difficult to obtain a large sample size to study genetic data. In this review, it was observed studies that looked at polymorphisms with sample sizes ranging from 35 to 647 children [24,31].

Different biological pathways have also been studied with respect to DNA methylation (Table 4 and Figure 1), but associations such as protection or susceptibility to OM have not yet been established. However, studies have shown that *DNMT1* methylation and hypomethylation in total DNA and in the *TNF-α* gene are associated with recovery of the oral mucosa. These data are quite interesting because since the epigenetic profile is modifiable, and if changes in the DNA methylation profile are necessary for mucosal healing, this opens a window of opportunity for studies on epidrugs (epigenetic drugs) for the treatment of oral mucositis, as they are already used to treat other diseases, such as cancer for example [60]. This can be very important for patients who develop a severe form of mucositis, as it significantly affects the patient’s quality of life.

For future studies, it is important to mention the chemotherapy regimen as well as the OM diagnosis scale (which was not found in all studies in this review). It is also important that other ethnicities are studied to obtain more accurate and representative data for a population, as allele frequencies can vary between populations. In addition, technologies such as customised high-throughput SNP chip arrays or genome sequencing can reveal the large-scale genetic profile of patients. Specifically, regarding DNA methylation, in addition to exploring other biological pathways, it is better to use oral mucosal cells rather than blood cells, as the methylation profile is site-specific and the inflammation is in the oral mucosa. It was noted that research into the epigenetics of oral mucositis is still in its infancy. In this sense, other mechanisms such as histone modifications and the expression of non-coding RNAs should also be investigated. Longitudinal studies could also contribute to the understanding of the epigenetic profile before and after chemotherapy.

Oral mucositis is one of the most common toxicities associated with cytotoxic cancer therapy and remains a significant burden for patients undergoing chemotherapy. The perspective is that the identification of genetic and epigenetic markers associated with oral mucositis will serve as the basis for implementing preventive screening protocols, using strategies to modify the dosage of chemotherapy or preventive therapies. However, it is important to note that many agents that can prevent mucositis are still being researched, and an important point is that prevention is better than curing.

Therefore, it is important that more genetic and epigenetic targets are studied in larger populations and in different ethnicities to validate a panel of genes associated with OM to try to identify patient susceptibility, design a strategy for prevention to reduce suffering and optimise treatment in these patients. As epigenetic markers are reversible, they may not only be potential susceptibility markers, but also markers of exposure to chemotherapy and targets for mucosal recovery therapies. A patient’s demographic, clinical, genetic and epigenetic data together contribute to precision medicine.

## Figures and Tables

**Table 1:** Instruments used for diagnosing and classifying the severity of oral mucositis.

Scale	Gradation	Characteristics
	Grade 1	Normality of voice, swallowing ability, texture of lips, tongue, jugal mucosa, palate, lip mucosa, gums and saliva consistency
**OAG** **(modified)**	Grade 2	Hoarse voice, pain on swallowing, dry/cracked lips, apparently shiny tongue with or without papillae and redness, buccal mucosa, palate and labial mucosa reddish/white but without ulceration, swollen gums with or without redness and thick saliva consistency/viscous
	Grade 3	Difficulty speaking/pain, inability to swallow, swollen/bleeding lips, blistered/cracked tongue, buccal mucosa, palate and labial mucosa with ulcers with or without bleeding, bleeding gums with or without stimulation and lack of saliva
	Grade 0	Without changes
	Grade 1	Erythema, irritations, pain
**WHO**	Grade 2	Erythema, ulcers (solid diet)
	Grade 3	Ulcers (liquid diet)
	Grade 4	Inability to feed
	Grade 0	Without changes
	Grade 1	No symptoms or mild symptoms, intervention not indicated
**NCI-** **CTCAE**	Grade 2	Moderate pain or ulcers that do not interfere with swallowing, diet modification indicated
	Grade 3	Severe pain interfering with swallowing
	Grade 4	Life-threatening consequences, urgent intervention indicated
	Grade 5	Death

OAG: Oral Assessment Guide; WHO: World Health Organization; NCI-CTCAE: National Cancer Institute’s-Common Terminology Criteria.

**Table 2:** Data from studies involving genetic polymorphisms and chemoinduced oral mucositis in paediatric patients with haematological malignancies.

Country Year [Reference]	Underlying Disease/ Diagnosis of OM	Sample	Chemotherapy Regimen (Includes MTX?)	Gene-Polymorphism	Technique/ Tissue	Outcome and Conclusion
Egypt 2010 [17]	Leukaemia/ No scale informed	40	Yes, with HD	*MTHFR* rs1801131, rs1801133	PCR-RFLP/Blood	*MTHFR* rs1801133 TT genotype was associated with susceptibility to OM
China 2011 [18]	Leukaemia/ CI-CTCAE	181	Yes	*MTHFR* rs1801131, rs1801133	PCR-RFLP/Blood	No association with OM
Slovenia 2011 [19]	Leukaemia or lymphoma/ WHO	64	Yes, with HD	*SLC19A1* rs1051266, *MTHFR* rs1801133, rs1801131, *MS* rs1805087, *MTRR* rs1801394, *TYMS* rs34743033, *ABCB1* rs2032582, rs1045642	PCR-RFLP, AS-PCR or TaqMan/Blood	*MTHFR* rs1801133 TT genotype was associated with susceptibility to OM; *TYMS* rs34743033 3R3R genotype was a protective factor for OM
Turquia 2012 [20]	Leukaemia/ WHO	115	Chemotherapy regimen not mentioned	*ABCB1* rs1045642	PCR-RFLP/Blood	*ABCB1* rs1045642 CT genotype was associated with susceptibility to OM
China 2013 [21]	Leukaemia/ NCI-CTCAE	164	Yes	*FPGS* rs1544105	PCR-RFLP/Blood	No association with OM
China 2014 [22]	Leukaemia/ NCI-CTCAE	112	Yes, with HD	*ABCC2* rs717620, rs3740065, *ABCC4* rs9516519, rs868853, rs2274407 *ABCG2* rs2231137	Microarray/ Blood	*ABCC2* rs717620 T allele was associated with susceptibility to OM
Spain 2014 [23]	Leukaemia/ WHO adapted	152	Yes, with HD	118 SNPs in 21 genes involved in the biogenesis and processing of miRNA	PCR-TaqMan/Blood	No association with OM
Mexico 2015 [24]	Leukaemia/ WHO and NCI-CTCAE	35	Yes	*XO* rs1701368, rs17323235 *ABCB1* rs1128503 *ABCC5* rs9838667, rs3792585	PCR-TaqMan/ tissue not mentioned	No association with OM
Mexico 2016 [25]	Leukaemia/ No scale informed	109	Yes, with HD	*ABCB1* rs1045642 *MTHFR* rs1801133	PCR-RFLP/Blood	*MTHFR* rs1801133 CC genotype was associated with susceptibility to OM
China 2017 [26]	Leukaemia/ NCI-CTCAE	322	Yes, with HD	12 SNPs in 4 genes: *SLCO1B1*, *SLC19A1*, *ABCB1*, *ABCG2*	PCR- Mass spectrometry/bone marrow	No association with OM
Dutch 2017 [27]	Leukaemia/ NCI-CTCAE	117	Yes	*CNOT4* rs3812265, *miR-1206* rs2114358, *miR-2053* rs10505168	AS-PCR and Taqman/Blood	*miR-1206* rs2114358 GG genotype was associated with susceptibility to OM
Dutch 2018 [28]	Leukaemia/ NCI-CTCAE	117	Yes, with HD	*TYMS* rs34743033, rs2853542, rs151264360	PCR-RFLP/Blood	No association with OM
Spain 2018 [29]	Leukaemia/ WHO adapted	179	Yes	213 SNPs in 206 genes of miRNAs	Microarray/ Blood or bone marrow	*miR-3683* rs6977967 GG genotype was associated with susceptibility to OM; *miR-4268* rs4674470 AG/GG genotypes were protective factors for OM
Serbia 2020 [30]	Leukaemia/ NCI-CTCAE	148	Yes, with HD	11 SNPs in 5 genes: *TYMS*, *MTHFR*, *DHFR*, *SLC19A1*, *SLCO1B*	ASO-PCR and sequencing/ tissue not mentioned	No association with OM
China 2021 [31]	Leukaemia/ NCI-CTCAE	647	Yes, with HD	28 SNPs in 14 genes: *ABCG2*, *ABCB1*, *ABCC1*, *ABCC2*, *ABCC4*, *SLCO1B*, *SLCO1A2*, *SLC19A1*, *MTHFR*, *ATIC*, *DHFR*, *GGH*, *FPGS*, *CCND1*	PCR and sequencing/Blood	*SLCO1B1* rs2306283 AG/GG genotypes were associated with susceptibility to OM
China 2021 [32]	Leukaemia or lymphoma/ NCI-CTCAE	80	Yes, with HD	23 SNPs in 15 genes: *SLC28A2*, *SLCO1B1*, *ABCB1*, *ABCC2*, *ABCC4*, *ABCG2*, *FPGS*, *GGH*, *MTHFR*, *MTHFD1*, *MTR*, *MTRR*, *GSTP1*, *ATIC*, *CCND1*	PCR- Mass spectrometry/Blood	*ABCB1* rs1128503 T, rs1045642 T, *MTHFR* rs1801133 T alleles were associated with susceptibility to OM
China 2021 [33]	Lymphoma/ NCI-CTCAE	63	Yes, with HD	*MTHFR* rs1801133, *SLCO1B1* rs11045879, rs4149056	PCR-HRM and TaqMan/ tissue not mentioned	No association with OM
China 2021 [34]	Lymphoma/ NCI-CTCAE	93	Yes, with HD	*MTHFR* rs1801133, rs1801131	PCR and sequencing/ tissue not mentioned	*MTHFR* rs1801133 CT/TT genotypes were associated with susceptibility to OM
Brazil 2021 [35]	Leukaemia/ OAG	64	Yes	*MTHFR* rs1801133, *DNMT3B* rs2424913, *ABCC2* rs717620, *ABCG2* rs2231137, rs2231142	PCR-RFLP/ Oral mucosa	*ABCG2* rs2231142 CA genotype were associated with susceptibility to OM
Brazil 2022 [36]	Leukaemia or lymphoma/ OAG	95	Yes	*SOD2* rs4880, *CAT* rs7943316, TNF-α rs1800629, *IL6* rs1800795	PCR-RFLP/ Oral mucosa	*CAT* rs7943316 AA genotype was associated with susceptibility to OM
Brazil 2023 [37]	Leukaemia or lymphoma/ OAG	102	Yes	*VDR* rs1544410, rs2228570, rs731236	PCR-RFLP/ Oral mucosa	*VDR* rs1544410 G allele and rs2228570 CT genotype were associated with susceptibility to OM
Brazil 2023 [38]	Leukaemia or lymphoma/ OAG	102	Yes	*DNMT1* rs2228611, *DNMT3A* rs7590760, *DNMT3B* rs6087990	PCR-RFLP/ Oral mucosa	No association with OM

OM: oral mucositis; MTX: methotrexate; HD: high dose; NCI-CTCAE: National Cancer Institute’s-Common Terminology Criteria.; WHO: World Health Organisation; OAG: Oral Assessment Guide; PCR-RFLP: Polymerase Chain Reaction-Restriction fragment length polymorphism; AS-PCR: Allele-specific-Polymerase Chain Reaction; ASO-PCR: Allele-specific oligonucleotide-polymerase chain reaction; PCR-HRM: Polymerase Chain Reaction-High-Resolution Melting. *MTHFR*: methylenetetrahydrofolate; *MTRR*: 5-methyltetrahydrofolate homocysteine methyltransferase reductase; *TYMS* (also known as TS): thymidylate synthetase; *MTHFD1*: methylenetetrahydrofolate dehydrogenase; *MTR*: 5-methyltetrahydrofolate homocysteine methyltransferase; *MS*: 5-methyltetrahydrofolate homocysteine methyltransferase; *FPGS*: folylpolyglutamate synthase; *GGH*: gamma-glutamyl hydrolase; *DHFR*: dihydrofolate reductase; *SLC19A1*: solute carrier family 19 member 1; *SLC28A2*: solute carrier family 28 member 2; *SLCO1A2*: solute carrier organic anion transporter family member 1A2; *SLCO1B*: solute carrier organic anion transporter family member 1B1; *ABCB1*: ATP-binding cassette of subfamily B member 1; *ABCC1*: ATP-binding cassette of subfamily C member 1; *ABCC2*: ATP-binding cassette subfamily C member 2; *ABCC4*: ATP-binding cassette subfamily C member 4; *ABCC5:* ATP-binding cassette subfamily C member 5; *ABCG2*: ATP-binding cassette of subfamily G member 2; *miR*: micro-RNA; *XO* (also known as XDH): xanthine dehydrogenase; *ATIC*: 5-aminoimidazole-4-carboxamide ribonucleotide formyltransferase/IMP cyclohydrolase; *CCND1*: cyclin D1; *CNOT4:* CCR4-NOT transcription complex subunit 4; *GSTP1*: glutathione S-transferase pi 1; *IL6*: interleukin 6; *TNF-α*: tumour necrosis factor alpha; *SOD2*: superoxide dismutase 2; *CAT*: catalase; *VDR*: vitamin D receptor; *DNMT*: DNA methyltransferase.

**Table 3:** Data from studies involving DNA methylation and chemoinduced oral mucositis in paediatric patients with haematological malignancies.

Country Year [Reference]	Underlying Disease/ Diagnosis of OM	Sample	Chemotherapy Regimen (Includes MTX?)	Methylation	Technique/Tissue	Outcome and Conclusion
The Netherlands 2018 [44]	Leukaemia/ NCI-CTCAE	82	Yes, with HD	Global (LINE-1)	PCR- Mass spectrometry/ Blood	No association with OM
Brazil 2023 [37]	Leukaemia or lymphoma/ OAG	81	Yes	Site-specific in *VDR* gene	MSP/ Oral mucosa	No association with OM
Brazil 2023 [38]	Leukaemia or lymphoma/ OAG	85	Yes	Site-specific in *DNMT1*, *DNMT3A*, *DNMT3B* genes	MSP/ Oral mucosa	*DNMT1* methylation was associated with mucosal recovery
Brazil 2024 [45]	Leukaemia or lymphoma/ OAG	85	Yes	Site-specific in *CAT*, *SOD3*, *IL-6*, *TNF-α* genes	MSP/ Oral mucosa	*TNF-α* hypomethylation was associated with mucosal recovery
Brazil 2024 [46]	Leukaemia/ OAG	76	Yes	Global, site-specific in *miR-9-1*, *miR-9-3* genes	ELISA, MSP/ Oral mucosa	Lower global methylation levels were associated with mucosal recovery

OM: oral mucositis; MTX: methotrexate; HD: high dose; NCI-CTCAE: National Cancer Institute’s-Common Terminology Criteria; OAG: Oral Assessment Guide; PCR: Polymerase Chain Reaction; MSP: methylation specific PCR; ELISA: Enzyme-Linked Immuno Sorbent Assay; LINE-1: long interspersed nucleotide element-1; *IL6:* interleukin 6; *TNF-α*: tumour necrosis factor alpha; *SOD3:* superoxide dismutase 3; *CAT*: catalase; *VDR*: vitamin D receptor; *DNMT*: DNA methyltransferase; *miR-9-1*: microRNA-9-1; *miR-9-3*:microRNA-9-3.

**Table 4:** Biological pathways investigated in oral mucositis in paediatric patients with haematological malignancies.

Pathway	Targets	Country
**Folic acid metabolism**	*MTHFR* ^2^, *MTRR*, *TYMS* ^1^, *MTHFD1*, *MTR*, *MS*, *FPGS*, *GGH*, *DHFR*	Egypt, China, Slovenia, Mexico, Dutch, Serbia, Brazil
**Transport proteins**	*ABCB1* ^2^, *ABCC1*, *ABCC2* ^2^, *ABCC4*, *ABCC5*, *ABCG2* ^2^, *SLC19A1*, *SLC28A2*, *SLCO1A2*, *SLCO1B* ^2^	Slovenia, Turquia, China, Mexico, Serbia, Brazil
**Epigenetic machinery**	*miR* ^1,2^, *global methylation* ^3^, *DNMT1* ^3^, *DNMT3A*, *DNMT3B*	Spain, Dutch, Netherlands, Brazil
**Other pathways** (purine metabolism, cell cycle, transcriptional regulator, detoxification, inflammation, oxidative stress, vitamin D metabolism)	*XO*, *ATIC*, *CCND1*, *CNOT4*, *GSTP1*, *IL6*, *TNF-α* ^3^, *VDR* ^2^, *SOD2*, *SOD3*, *CAT* ^2^	Mexico, Dutch, China, Brazil

In total, 402 polymorphisms were analysed in 259 genes (most of them were microRNA genes, here referred to as *miR*); for DNA methylation, global methylation and site-specific methylation were analysed in 10 genes. Most of the data on polymorphisms came from China (36%), followed by European (27%) and Brazilian (18%) studies. In terms of DNA methylation, the largest contribution comes from Brazilian studies (80%), followed by the Netherlands (20%). ^1^ targets associated with protection from OM; ^2^ targets associated with susceptibility of OM; ^3^ targets associated with mucosal recovery.

## Data Availability

All the data contained in the manuscript will be made available from the corresponding author upon reasonable request.
